# Composition and Electronic Structure of La_2_O_3_/CNFs@C Core-Shell Nanoparticles with Variable Oxygen Content

**DOI:** 10.3390/nano13222945

**Published:** 2023-11-14

**Authors:** Evgeniya V. Suslova, Alexander N. Ulyanov, Alexey P. Kozlov, Denis A. Shashurin, Serguei V. Savilov, Georgy A. Chelkov

**Affiliations:** 1 Department of Chemistry, Lomonosov Moscow State University, 119991 Moscow, Russiakozlov.aleksei.p@gmail.com (A.P.K.);; 2 Faculty of Medicine, Lomonosov Moscow State University, 119991 Moscow, Russia; 3 Joint Institute for Nuclear Research, 141980 Dubna, Russia; chelkov@jinr.ru

**Keywords:** lanthanum, carbon nanoflakes, core-shell, gas-phase oxidation, electronic structure, electron paramagnetic resonance

## Abstract

La_2_O_3_ nanoparticles stabilized on carbon nanoflake (CNF) matrix were synthesized and graphitized to produce core-shell structures La_2_O_3_/CNFs@C. Further oxidation of these structures by nitric acid vapors for 1, 3 or 6 h was performed, and surface-oxidized particles La_2_O_3_/CNFs@C_x (x = 1, 3, 6) were produced. Bulk and surface compositions of La_2_O_3_/CNFs@C and La_2_O_3_/CNFs@C_x were investigated by thermogravimetric analysis and X-ray photoelectron spectroscopy. With increasing the duration of oxidation, the oxygen and La_2_O_3_ content in the La_2_O_3_/CNFs@C_x samples increased. The electronic structures of samples were assessed by electron paramagnetic resonance. Two paramagnetic centers were associated with unpaired localized and mobile electrons and were registered in all samples. The correlation between bulk and surface compositions of the samples and their electronic structures was investigated for the first time. The impact of the ratio between *sp*^2^- and *sp*^3^-hybridized C atoms, the number and nature of oxygen-containing groups on the surface and the presence and proportion of coordinated La atoms on the EPR spectra was demonstrated.

## 1. Introduction

Different lanthanum and lanthanide-containing compounds deposited on the carbon nanomaterials (CNMs) are currently considered promising materials for contrast agents for magnetic resonance imaging (MRI) [1,2] and computed tomography [3,4,5]. Oxide La_2_O_3_, in combination with reduced graphene oxide (rGO), is an effective catalyst for the synthesis of dimethyl carbonate [6] and bis(indolyl)methanes [7]. La_2_O_3_/rGO and La_2_O_3_ supported on carbon nanotubes (CNTs) can be used for energy storage devices, including asymmetric supercapacitors [8,9]. La_2_O_3_ encapsulated into the CNT cavity is a felicitous composite material for Pt-free counter electrodes of solar cells [10].

CNMs are unique supports because their surface properties depend on the allotropic modification of carbon, particle size, surface defectiveness, functional groups, etc. The CNM surface can be easily modified by hydroxyl and carboxyl groups, resulting in further coordination of Ln^3+^ (or other metal ions) with the formation of stable chelate complexes [11,12]. Nanodiamonds (NDs), carbon nanoflakes (CNFs) or graphene nanoflakes (GNFs), CNTs and nitrogen-doped carbon dots are described as supports of Ln^3+^ as well [1,3,5,13,14]. An interesting example of Ln/CNM composites is the endohedral fullerenes with a Ln@C_n_ structure that purports the encapsulation of Ln^3+^ ions or Ln metal atoms inside the fullerene sphere [15,16,17]. Ln^3+^ ions are coordinated with oxygen atoms to result in the C-O-Ln bond formation. The M^n+^ ions are typically coordinated by two surface functional groups that are coordinately saturated with solvent molecules and anions that were originally present in the metal precursor salt [12,14]. It was, for example, shown using HRTEM in HAADF mode and EPS spectra that Tm^3+^ ions uniformly distribute on the oxidized CNT surface and mainly at the edges after thermal defunctionalization of CNTs [13].

Electron paramagnetic resonance (EPR) is a suitable method for the study of compounds and materials with free electrons, radicals, radical ions, particles in the triplet state, as well as paramagnetic ions of transition and rare earth metals. To summarize, both CNMs and lanthanide derivatives are suitable objects for EPR studies [17,18,19,20]. The CNTs [21,22], nitrogen-doped CNTs [23], boron-doped CNTs [24], CNFs [25,26,27], consolidate of CNTs and CNFs [28,29,30,31], GO [32,33,34], graphene [35], GO aerogels [36,37], GO quantum dots [38], carbon nanofibers [39], glass-like carbon [40], detonation diamonds and NDs [12], graphite-polystyrene composite [41], carbon films [42], natural coals [43], oxidized coals [44], carbon blacks [45], different carbon composites [46] and others [18] were investigated using the EPR. EPR signals contain information about possible paramagnetic centers of CNMs, their nature and the possible influence of oxygen-containing groups [28,47,48,49]. The temperature dependencies of the intensity of the EPR spectra are used to identify the source of the observed magnetism [34]. For example, a specific π-electronic zone, stabilized by saddle-shaped edges of nanographene, was at first predicted theoretically and then was demonstrated by the EPR method [50,51]. The EPR can be used to study the mechanism of reactions on the surface of CNMs. For example, it has been proven that F^-^ ion reacted region-selectively with the C_70_ fullerene [52].

The most often rare earth elements and their compounds studied by EPR are gadolinium derivatives [20,53,54] because only ions Gd^3+^ and Eu^2+^ EPR spectra at room temperature can be observed [19]. For some Ln derivatives, for example, for Ln(OH)_3_ (Ln = La, Pr, Nd), EPR spectra can provide information about oxygen vacancies and the coordination environment of Ln^3+^ [55,56,57].

Panich, Osipov and other authors described the EPR spectra of different complexes M^n+^/CNMs where CNMs are ND or detonation diamond with metal ions Cu^2+^, Co^2+^, Gd^3+^, Fe^2+^, Fe^3+^ and Mn^2+^ [11,12,58,59,60,61]. It has been shown that the EPR allows distinguishing signals from two types of spins: the carbon spins localized inside the ND particles and the spins of M^n+^ ions on the surface.

In the present work, we observe and discuss the properties of La_2_O_3_/CNFs@C nanosized composite assessed by EPR and XPS. The 2–3 nm La_2_O_3_ particles deposited on the CNF surface and encapsulated with the shells from 2–3 graphene layers were studied. This material is used as a contrast agent for computed tomography [3,5]. The CNF matrix is necessary for 2–3 nm of La_2_O_3_ particles’ stabilization, preventing their agglomeration and enlargement. Graphene shells allow the functionalization of the particle surface without affecting the La_2_O_3_ core, modifying their chemical and pharmacological properties without impacting their roentgenological characteristics. Specifically, oxidative treatment of the surface leads to its modification with carboxyl and hydroxyl groups. These groups can be subsequently used to decorate the surface with various functional groups or small molecules with high affinity to specific biopolymers, forming contrasts with high selectivity to the structures of interest. Such synthetic work requires a comprehensive understanding of the composite structure and properties as well as their correlation with various factors such as duration or degree of oxidation. So, we studied the composites that underwent the post-functionalization of graphene shells with carboxyl and hydroxyl groups by HNO_3_ vapors during 1.0, 3.0 and 6.0 h. The different durations of oxidation treatment of La_2_O_3_/CNFs@C resulted in the different degrees of graphene shell degradation and oxygen content and change in its electronic structure. The oxidized materials were investigated by EPR to establish how the surface and volume content of La_2_O_3_, as well as the number and nature of oxygen-containing groups, influence the electronic structure of particles. The changes in composition and structure, as well as the increase in oxygen-containing groups, had the most dramatic effect on the EPR spectra. The intensity, width of spectra and number of unpaired electrons were found to be different.

## 2. Materials and Methods

### 2.1. Synthesis

**Figure 1:**
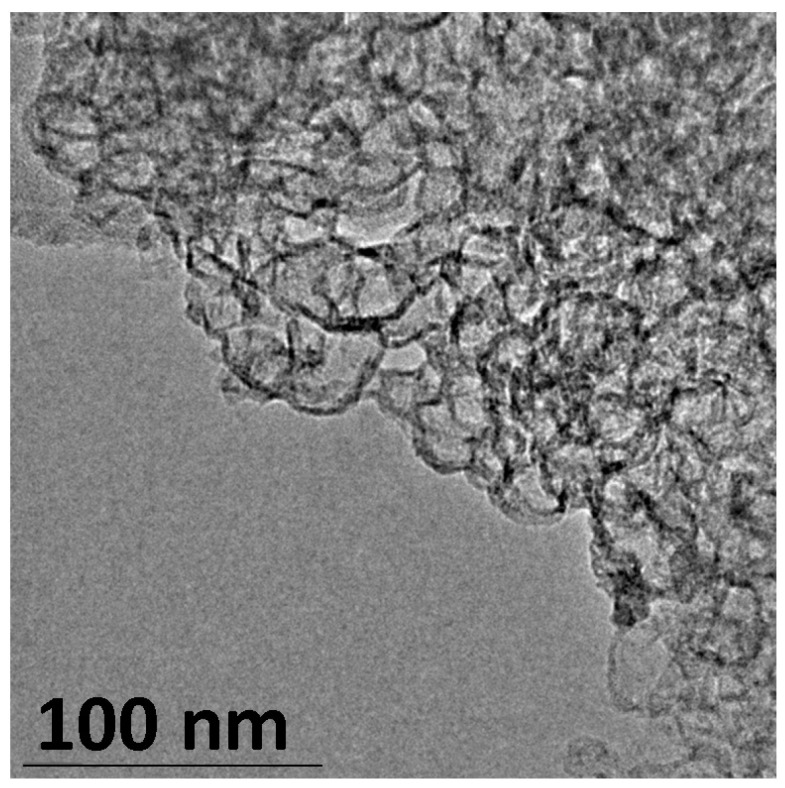
TEM image of raw CNFs.

Carbon nanoflakes were synthesized by pyrolysis of hexane (99.8%, Reachim) at 900 °C in the presence of MgO template [62]. MgO was dissolved in boiling 34% HCl (99.9%, Reachim) solution. Obtained CNFs were washed with dt. water and dried at 110 °C. The CNF particles replicated the template’s form and consisted of 7–15 graphene layers (Figure 1). The subsequent surface oxidation of CNFs was carried out with boiling 67–69% nitric acid (Component-Reaktiv) solution for 1 h for the most uniform subsequent distribution of La^3+^ ions [3,13].

**Figure 2:**
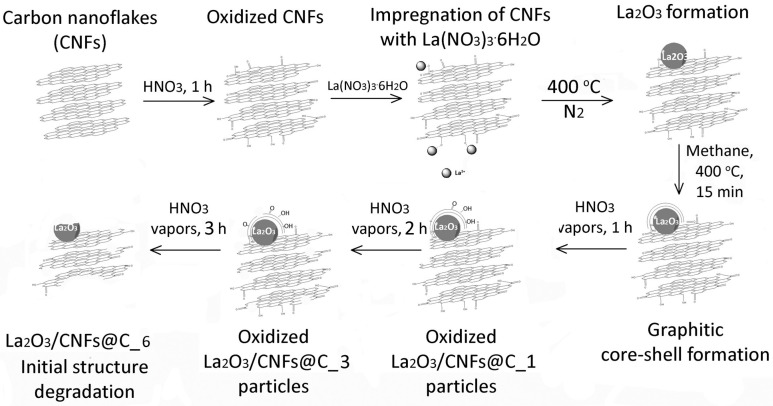
The scheme of synthesis processes.

The synthesis, properties and characteristics of 2–3 nm sized La_2_O_3_ particles stabilized with CNF matrix (La_2_O_3_/CNFs) have been described in detail in our earlier works [3,5]. The La_2_O_3_/CNF composite was prepared by impregnation of 0.10 g oxidized CNFs with 0.146 g of La(NO_3_)_3_·6H_2_O (99%, China Northern Rare Earth Group High-Tech Co., Ltd., Baotou, China) in the 100 mL ethanolic (99.99%, Merck, Darmstadt, Germany) solution with further solvent evaporation and decomposition of nitrate at 400 °C under nitrogen (99.999%, Logika Ltd., Moscow, Russia) flow. Core-shell particles La_2_O_3_/CNFs@C have been prepared by encapsulation of La_2_O_3_/CNFs with the graphene shells via methane (99.99% Moscow Gas Processing Plant, Moscow, Russia) decomposition at 400 °C [63]. Gas-phase oxidation of La_2_O_3_/CNFs@C was described elsewhere [64]. The La_2_O_3_/CNFs@C sample was placed in an open box in an atmosphere of boiling nitric acid with a temperature of 83 °C and kept for 1.0, 3.0 or 6.0 h. The vapor phase was a complex mixture of HNO_3_, O_2_, H_2_O and various nitrogen oxides [65]. Then, the oxidized samples La_2_O_3_/CNFs@C_x (where x is the duration of oxidation equal to 1, 3 or 6) were washed with water to delete nitric acid and partially dissolved La-containing fraction. The scheme illustrating and explaining the sequence of synthesis procedures is shown in Figure 2.

### 2.2. Physico-Chemical Analysis

Thermogravimetric (TG) analysis was performed on a Netzsch STA 449 PC LUXX instrument (Netzsch, Selb, Germany) with a sample heating rate of 5 °C·min^−1^ and a temperature range of 25–1000 °C in an air atmosphere. The error of the unburned impurity definition was less than 2%.

Transmission electron microscopy (TEM) images were recorded on a JEOL 2100F/Cs (Jeol, Tokyo, Japan) microscope operated at 200 kV and equipped with a UHR pole tip, a spherical aberration corrector (CEOS, Heidelberg, Germany) and an EEL spectrometer (Gatan, Germany).

The X-ray photoelectron spectra (XPS) were recorded on an Axis Ultra DLD spectrometer (Kratos Analytical, Milton Keynes, UK) using monochromatic AlKα radiation (1486.7 eV). The pass energies of the analyzer were 160 eV for survey spectra and 40 eV for high-resolution scans. The error in element definition was about 5%.

Electron paramagnetic resonance (EPR) measurements were performed with a BRUKER EMX 6/1 spectrometer at 9.8–9.9 GHz (X-band) at room temperature. The spectra of studied and standard/reference samples were recorded at the microwave power of 0.635 mW (well below the saturation power of ≈2.0 mW), modulation amplitude of 1.0 G, modulation frequency of 100.00 kHz, time constant of 40.960 ms, sweep time of 335.54 s, number of scans of 1 and receiver gain equal to 1.78 × 10^3^. The EPR measurements of the studied as well as of standard samples were performed in the ampule with the outer and inner diameters of 3.9 and 2.8 mm, respectively. TEMPO (2,2,6,6-tetramethyl-1-piperidinyloxyl) in toluene solution was used as a reference sample with the number of paramagnetic centers *N*_st_ = 2.0792 × 10^16^ spins. The height of the studied and reference samples in the ampule was about 5.5 mm. The *N*-value for the studied samples was deduced using the expression *N* = (Q_st_/Q)·(*DI*·*N*_st_)/*DI*_st_, where Q_st_ and Q are the quality factors of the resonator with the reference and studied samples, respectively, and *DI* and *DI*_st_ are double integrated intensities of the resonance spectrum limes of the studied and reference samples, respectively. The measurements were performed using samples with almost equal mass (about 8 mg). The *DI* values were deduced by the integration of the initial raw experimental spectra with the consequent normalization of the sample mass. The Q/Q_st_ ratio was ≈0.9. The background signal was subtracted using the Bruker WinEPR System Version 2.11b software.

## 3. Results and Discussion

The morphology and particle size were investigated by TEM and reported earlier [63,64]. According to TEM images, the La_2_O_3_ particles with 2–3 nm size are uniformly distributed on the CNF surface. Graphene shells contain 2–3 layers forming a La_2_O_3_/CNFs@C composite with a core-shell structure. After gas-phase oxidation, the carbon particles degraded with partial loss of initial structure (Figure 2) [63,64].

**Figure 3:**
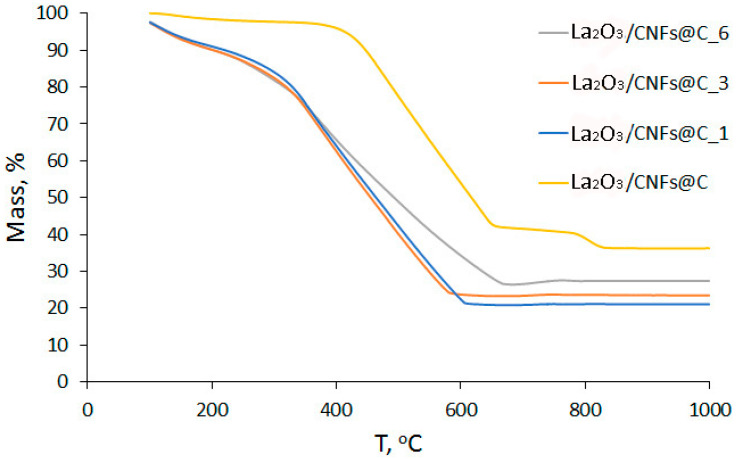
TG curves of La_2_O_3_/CNFs@C and La_2_O_3_/CNFs@C_x (x = 1, 3, 6) samples.

The La_2_O_3_ bulk content in the composition of samples La_2_O_3_/CNFs@C and La_2_O_3_/CNFs@C_x (x = 1, 3, 6) was confirmed by thermogravimetric (TG) analysis through the determination of the unburned impurities (Figure 3). According to TG data, the La_2_O_3_ content in the La_2_O_3_/CNFs@C_x was 21.08, 23.48 and 27.39 wt. % for x = 1, 2 and 3, respectively. The La_2_O_3_ content in the La_2_O_3_/CNFs@C was 36.24 wt. %. This indicates that the increase in the duration of the oxidation was accompanied by a partial loss of the carbon component in the samples.

**Figure 4:**
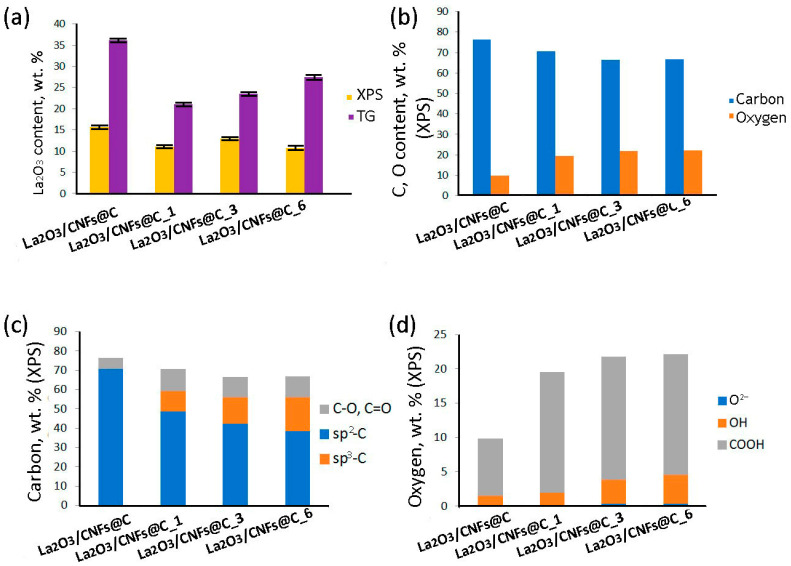
TG and XPS data correspond to bulk and surface La_2_O_3_ content (**a**). Carbon and oxygen content according to XPS (**b**). The ratio between different carbon (**c**) and oxygen (**d**) atom types according to XPS.

**Figure 5:**
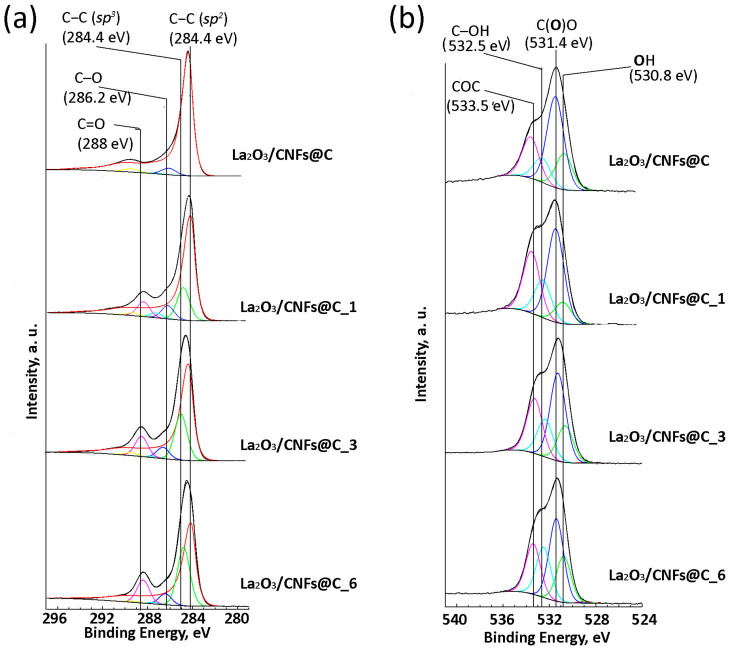
Survey C1s (**a**) and O1s (**b**) XPS spectra of La_2_O_3_/CNFs@C and La_2_O_3_/CNFs@C_x samples.

The content of La_2_O_3_ in the samples is different for the samples with different x (Figure 4a) according to both TG (indicating the bulk composition of the sample) and XPS (reflecting the composition of approximately 10 upper layers of the surface). The extent and direction of the differences vary between TG and XPS, demonstrating different impacts of extension of the oxidation to the bulk and the surface of the samples. According to XPS data, the oxygen and carbon content differ as well. The amount of carbon decreased with the increase in oxidation duration, and oxygen understandably increased (Figure 4b). According to the C1s XPS spectra, the oxidized samples La_2_O_3_/CNFs@C_x have *sp*^2^- and *sp*^3^-hybridized carbon atoms and oxygen-bonded carbon species (Figure 5a) [64]. The O1s XPS spectra show that oxygen can present as O^2−^ in the La-O fragments, lanthanum-bonded hydroxyl groups (OH^−^), and different oxygen species, C–O, C=O, etc., bonded to carbon atoms (Figure 5b) [64]. The ratio between different carbon and oxygen forms changed with a change in gas-phase oxidation duration (Figure 4c,d).

**Figure 6:**
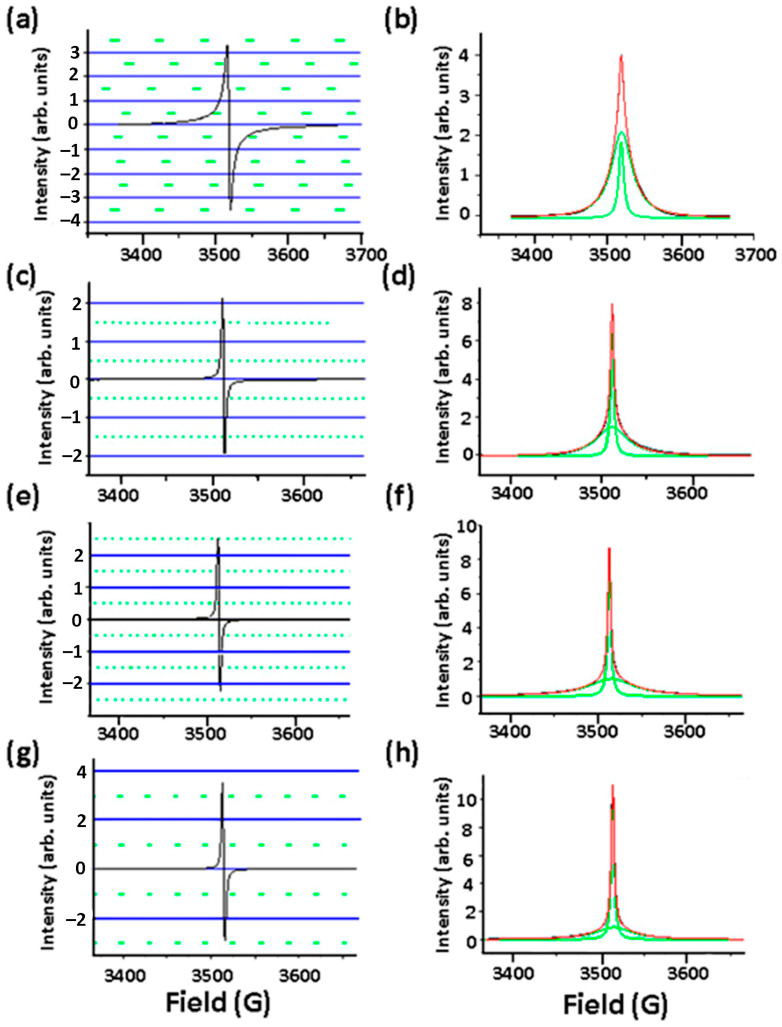
EPR spectra of La_2_O_3_/CNFs@C (**a**), La_2_O_3_/CNFs@C_1 (**c**), La_2_O_3_/CNFs@C_3 (**e**) and La_2_O_3_/CNFs@C_6 (**g**). Absorption EPR spectra of La_2_O_3_/CNFs@C (**b**), La_2_O_3_/CNFs@C_1 (**d**), La_2_O_3_/CNFs@C_3 (**f**) and La_2_O_3_/CNFs@C_6 (**h**). Background signal was subtracted using the Bruker WinEPR System Version 2.11b software. Absorption spectra were fitted with two Lorentzian lines.

Figure 6 shows the EPR spectra of La_2_O_3_/CNFs@C and La_2_O_3_/CNFs@C_x compositions. Initial raw spectra are presented in Figure 6a,c,e,g. Absorption spectra, obtained by the integration of the raw ones, are shown in Figure 6b,d,f,h. The asymmetry of the raw spectra is caused by the fine structure of the absorption ones. EPR spectra of all samples differ in width, positions (*g*-factor) and number of spins (*N*). The absorption spectra were simulated by Gaussian, Lorentzian and Voigt functions. The best fit was obtained with the Lorentzian. All observed spectra are well fitted with two Lorentzian lines with different widths corresponding to *g_l_*- and *g_h_*-factors and located at low- and high-applied fields, respectively (Figure 6b,d,f,h). Therefore, we consider two types of paramagnetic centers attributed to these lines [29,49].

**Figure 7:**
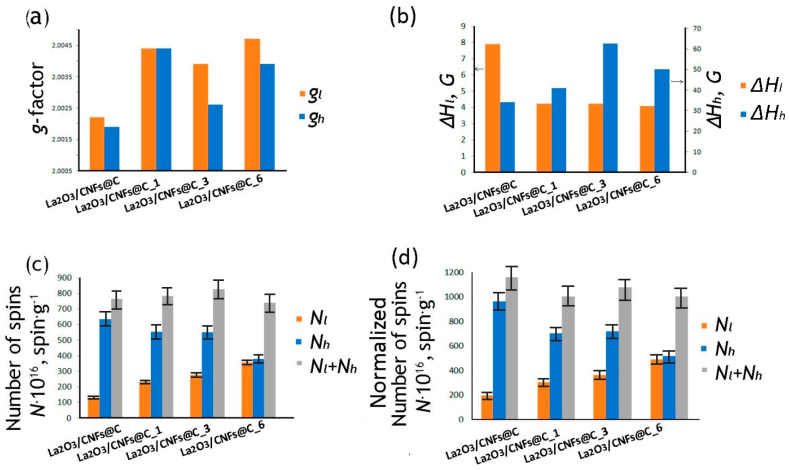
The changes of *g*-factors (**a**), linewidths (Δ*H*) (**b**), numbers of spins (*N*) (**c**) and normalized to carbon-oxygen frame (**d**) of EPR spectrum lines of samples. Subscripts *l* and *h* denote the lines attributed to the peaks, located at low and high applied field. The error in the Δ*H* determination is about 3%. The error of g-factor is about 0.0000(5). Uncertainty of the spin numbers is less than 15%.

EPR spectra of La^3+^ are typically silent because of the absence of unpaired electrons in its electron structure, which corresponds to [Xe]6s^0^5d^0^. Only La^2+^ ions and zero valence La atoms were characterized by EPR spectra at low temperatures [17,66]. In the EPR spectra of La(OH)_3_, the *g*-factor equaled 2.02788 and corresponded to oxygen vacancies in the La(OH)_3_ structure [55]. In the present study, both the *g_l_* and *g_h_* values are 2.0022–2.0047 (Figure 7a). There are no correlations observed between *g_l_* and *g_h_* and La^3+^ bulk and/or surface concentrations according to TG and XPS data in the La_2_O_3_/CNFs@C and La_2_O_3_/CNFs@C_x (x = 1, 3, 6), respectively (Figure S1). Due to this, we concluded that the EPR spectra were predominantly defined by the carbon matrix.

Two EPR lines (named broad and narrow as per standard terminology), associated with two *g*-factors and reflecting the presence of two different types of paramagnetic centers, were previously reported for different CNMs: GO [32,51], ND [67], CNFs, CNTs [25,29,30,31,47,48,50], etc. A broad line may be related to the defect states strongly coupled with itinerant spins and/or conduction electrons within the *sp*^2^-clusters [25,27,32,48,51,68]. The narrow line was attributed to the localized electrons provoked by terminal *sp*^3^-hybridized carbon atoms and various vacancies in the graphene layers [25,27,32,48,51,68,69]. These are the terminal energetically unsaturated carbon atoms combined with oxygen atoms and molecules, forming functional groups or saturated due to chemisorption. Only one line (*g*-factor is 2.0028) is present in the *sp*^3^-hybridized carbon atoms in the structure of the diamond. Paramagnetism of ~5 nm diamond is associated with intrinsic paramagnetic defects of the diamond crystal lattice with an unpaired electron located within the near-surface belt at the distance of 0.3–0.9 nm from the surface [70]. In the present study, the values of the *g*-factors are 2.0022–2.0047 (Figure 7a). The value of the *g_l_*-factor of oxidized CNFs is 2.0010 [25].

According to XPS data, the content of *sp*^3^-hybridized carbon atoms and carbon-oxygen bonds increased with the increase of oxidation duration (Figure 4c). We have also previously shown that an increase in the oxidation time of CNTs and CNFs, as well as consolidated and oxidized CNTs and CNFs, leads to an increase in oxygen groups and surface defectiveness according to Raman spectra [71] and, at the same time, the number of paramagnetic centers [25,28,29,31,48,49].

The width of EPR lines is highly sensitive to oxygen in the CNM compositions. Broadening of ∆*H_h_* lines has a satisfactory correlation with the content of surface oxygen in the samples La_2_O_3_/CNFs@C → La_2_O_3_/CNFs@C_1 → La_2_O_3_/CNFs@C_3 (Figure 7b) that is in line with the data [47,68]. Based on this, we concluded that the line at the high applied field corresponds to the unpaired electrons on the terminal *sp*^3^-hybridized carbon atoms. This was additionally confirmed by a decrease in the number of unpaired *N_h_* electrons (Figure 7c), caused by the increase of the C=O terminal groups that lead to the disappearance of the edge electrons and do not contribute to the EPR signal [47]. At the same time, the ∆*H_l_* line width did not change in the row La_2_O_3_/CNFs@C_1 → La_2_O_3_/CNFs@C_3 → La_2_O_3_/CNFs@C_6 (Figure 7b), while the content of unpaired electrons *N_l_* increased (Figure 7c). So, we concluded that the line at the low applied field corresponds to itinerant spins within the *sp*^2^-clusters.

**Figure 8:**
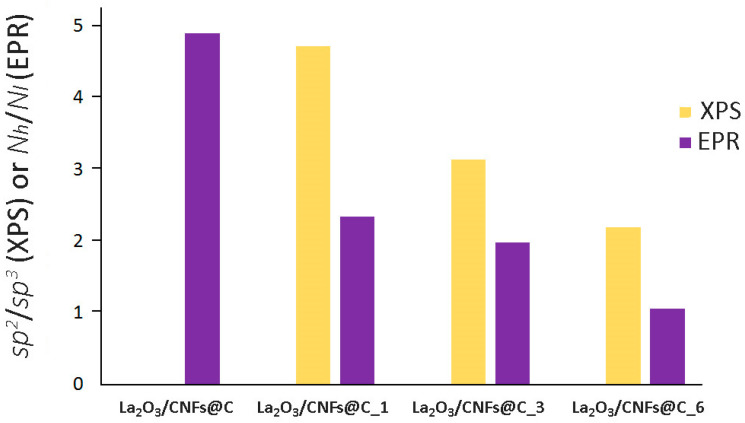
The ratio of *sp*^2^/*sp*^3^-hybridized carbon atoms and *N_h_*/*N_l_* according to XPS and EPR data.

The correlation between the ratio of *sp*^2^ and *sp*^3^-hybridized carbon atoms for samples according to XPS and EPR data is shown in Figure 8. The *sp*^2^/*sp*^3^ ratio (XPS) and *N_h_*/*N_l_* ratio (EPR) associated with *sp*^2^/*sp*^3^ decreased with an increase in the oxidation time according to both methods. The *sp*^2^/*sp*^3^ ratio for non-oxidized sample La_2_O_3_/CNFs@C could not be assessed by XPS due to the massive prevalence of *sp*^2^-hybridized carbon atoms. This ratio became assessable only after the oxidation of the sample. The *N_h_*/*N_l_* ratio could be assessed by EPR for all samples. This difference can be attributed to the higher impact of the oxidation towards the surface assessed by XPS and the preservation of the bulk sample assessed by EPR.

Another important factor affecting the EPR spectra of samples is the presence of La_2_O_3_ particles that are coordinated by the surface oxygen atoms and can also affect the electronic interaction in the system, similar to how high-polar carbon bonded with Fe^2+^ ions has its own manifestation in the ERP [72]. La^3+^ ions, coordinated by oxygen atoms with C-O-La bridge formation, are distributed over the CNF surface, mainly in the areas of localization of the oxygen-containing groups [13]. C-O-La fragments impact the surface electronic structure of the carbon matrix. To estimate the contribution of La^3+^, we normalized the number of spins only to the carbon-oxygen component (Figure 7d). The sum *N_l_* + *N_h_* increased (Figure 7c) with an increase of specific oxygen content in the composition of La_2_O_3_/CNFs@C, La_2_O_3_/CNFs@C_1 and La_2_O_3_/CNFs@C_3 samples. However, the recalculation of the number of unpaired electrons only on the carbon-oxygen component led to the decrease of *N_l_* + *N_h_* (Figure 7d). We believe that this trend was driven by a change in the structure of the graphene shells and CNFs caused by oxidation, i.e., significant degradation of graphene sheets due to etching with nitric acid [64]. Specifically, the oxidation during 6 h (La_2_O_3_/CNFs@C_6 sample) led to the strong damage of the carbon structure and change of the sample composition, including decrease of the carbon content (Figure 4b).

The effects of the size of the CNMs on the EPR signal were described in numerous studies [51,73,74]. It was shown that the decrease of ND size from 100 down to 18 nm led to the disappearance of the characteristic hyperfine splitting in the EPR spectrum and the appearance of the two lines [73]. The increase of the edged carbon atoms content resulted in the increase of unpaired electrons because of the fragmentation of rGO [51]. The CNF sizes in the samples were smaller than 18 nm, which allowed interlayer interaction, resulting in a high paramagnetic response. This is consistent with the statement that the interlayer interaction changes the nature of the π-wave function from 2D (single-layer) to 3D (volumetric) in multilayer graphene, which is associated with electron mobility and unique high sensitivity of the band structure and materials [75,76].

## 4. Conclusions

The electronic structure of La_2_O_3_/CNFs@C was studied. The EPR spectra of core-shell structured La_2_O_3_/CNFs@C and gas-phase oxidized La_2_O_3_/CNFs@C_x (where x = 1, 3 or 6 is the duration of oxidation) composites developed as potential contrast agents for computed tomography. La_2_O_3_/CNFs@C_x composition changed under gas-phase treatment with HNO_3_ vapors. With an increase in oxidation duration, the surface and bulk content of La_2_O_3_ in the La_2_O_3_/CNFs@C_x increased because of carbon shell oxidation.

The EPR spectra of all samples differed in intensity, width, positions (*g*-factor) and number of spins (*N*). Two *g_l_*- and *g_h_*-factors located at low- and high-applied fields, respectively, were determined for each sample. This indicates two types of paramagnetic centers. The spin density is associated with the lines and correlated with the spin number of localized and mobile electrons. The content of oxygen in the samples and, consequently, the content of localized unpaired electrons *N_l_* associated with terminal *sp*^3^-hybridized carbon atoms increased with an increase in the oxidation duration. In parallel, the content of unpaired electrons *N_h_* decreased. We conclude that the line at the low-applied field corresponded to itinerant spins within the *sp*^2^-clusters. It was also shown that the number of unpaired electrons in the La_2_O_3_/CNFs@C and La_2_O_3_/CNFs@C_x structure was influenced by many factors, including (1) the ratio between *sp*^2^- and *sp*^3^-hybridized C atoms, (2) the number and nature of oxygen-containing groups on the surface, and (3) the presence and proportion of coordinated La atoms because of differences of lanthanum content between TG and XPS data.

## Data Availability

Data are contained within the article and Supplementary Materials.

## Supplementary Materials

The following supporting information can be downloaded at: https://www.mdpi.com/article/10.3390/nano13222945/s1, Figure S1: Correlations between *g_l_*, *g_h_*, surface (TG) and bulk (XPS) La content.
