# Supplementary material for: Composition and Electronic Structure of La2O3/CNFs@C Core-Shell Nanoparticles with Variable Oxygen Content
Source: Nanomaterials (Basel). 2023 Nov 14;13(22):2945. doi: 10.3390/nano13222945 (PMC10674885; doi:10.3390/nano13222945)
Supplement: Supplementary file 1 [file nanomaterials-13-02945-s001.zip › nanomaterials-2698975-supplementary.pdf]

Supplementary materials

Composition and Electronic Structure of La_2O_3/CNFs@C Core-Shell Nanoparticles with Variable Oxygen Content

Evgeniya V. Suslova [1,*], Alexander N. Ulyanov [1], Alexey P. Kozlov [1], Denis A. Shashurin [2], Serguei V. Savilov [1] and Georgy A. Chelkov [3]

[1] Department of Chemistry, Lomonosov Moscow State University, 119991 Moscow, Russia
[2] Faculty of Medicine, Lomonosov Moscow State University, 119991 Moscow, Russia
[3] Joint Institute for Nuclear Research, 141980 Dubna, Russia
* Correspondence: suslova@kge.msu.ru

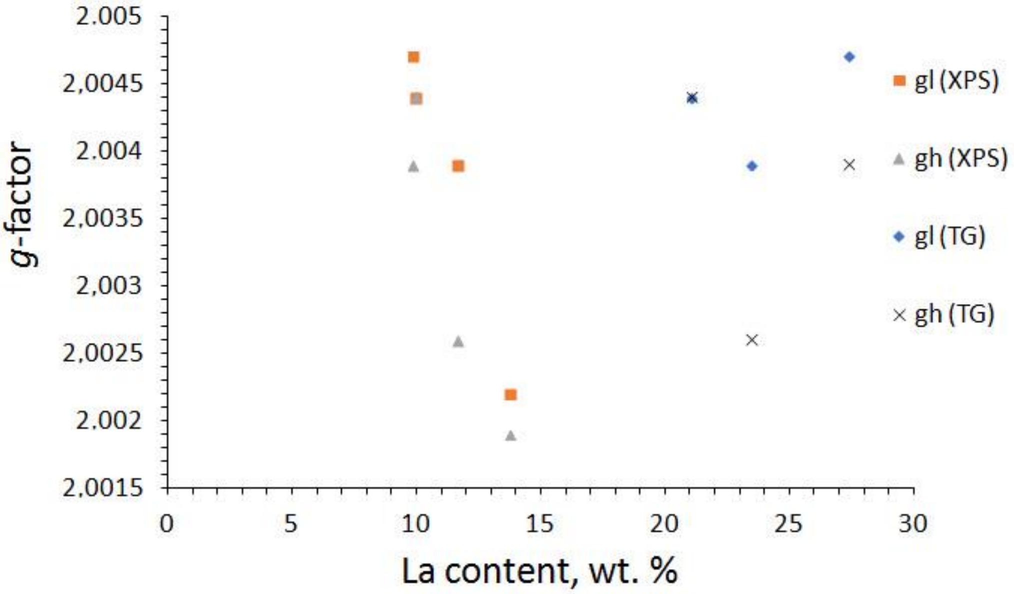

Figure S1. Correlations between g_l, g_h, surface (TG) and bulk (XPS) La content.
